# “Decision-making capacity for research participation among addicted people: a cross-sectional study”

**DOI:** 10.1186/s12910-015-0086-9

**Published:** 2016-01-13

**Authors:** Inés Morán-Sánchez, Aurelio Luna, Maria Sánchez-Muñoz, Beatriz Aguilera-Alcaraz, Maria D. Pérez-Cárceles

**Affiliations:** Mental Health Centre (Health Service of Murcia), Real St 8, E-30201, Cartagena (Murcia), Spain; Department of Legal and Forensic Medicine, Biomedical Research Institute (IMIB), Regional Campus of International Excellence “Campus Mare Nostrum”, Faculty of Medicine, University of Murcia, (Murcia), Spain; Isaac Peral Health Centre (Health Service of Murcia), Ulloa St 6. E 30300, Cartagena (Murcia), Spain

**Keywords:** Research Ethics, Mental Competency, Decision-making, Informed consent, Substance-Related Disorders

## Abstract

**Background:**

Informed consent is a key element of ethical clinical research. Addicted population may be at risk for impaired consent capacity. However, very little research has focused on their comprehension of consent forms. The aim of this study is to assess the capacity of addicted individuals to provide consent to research.

**Methods:**

53 subjects with DSM-5 diagnoses of a Substance Use Disorder (SUD) and 50 non psychiatric comparison subjects (NPCs) participated in the survey from December 2014 to March 2015. This cross-sectional study was carried out at a community-based Outpatient Treatment Center and at an urban-located Health Centre in Spain. A binary judgment of capacity/incapacity was made guided by the MacArthur Competence Assessment Tool for Clinical Research (MacCAT–CR) and a clinical interview. Demographics and clinical characteristics were assessed by cases notes and the Mini-Mental State Examination, the Global Assessment Functional Scale and the Clinical Global Impression Scale.

**Results:**

NPCs performed the best on the MacCAT–CR, and patients with SUD had the worst performance, particularly on the Understanding and Appreciation subscales. 32.7 % SUD people lacked research-related decisional capacity. There were no statistically significant differences between the groups in terms of capacity to consent to research.

**Conclusions:**

The findings of our study provide evidence that a large proportion of individuals with SUD had decisional capacity for consent to research. It is therefore inappropriate to draw conclusions about capacity to make research decisions on the basis of a SUD diagnosis. In the absence of advanced cognitive impairment, acute withdrawal or intoxication, we should assume that addicted persons possess decision-making capacity. Thus, the view that people with SUD would ipso facto lose decision-making power for research consent is flawed and stigmatizing.

## Background

Obtaining informed consent is a cornerstone of biomedical research. It constitutes a fundamental ethical requirement that is given priority in all national and international research ethics codes [1, 2]. Valid informed consent requires the researcher to ensure that the consent provided is voluntary and the patient is competent to make the decision [3]. The standard informed consent process of requiring a routine signature on a document does not ensure fully shared decision-making [4], it’s necessary to assess the capacity of research participants to provide meaningful consent prior to entry into clinical trials.

In Spain, there are no defined guidelines as to who should assess patient decision-making competence or how such assessments should be accomplished. Spanish laws about informed consent in biomedical research touch upon a subject's decision-making capacity and indicate those situations where the capacity is limited without defining or specifying how it should be assessed [5, 6]. Laws emphasise the necessity of justifying the inclusion of “vulnerable populations” in research, without specifying who these vulnerable populations are [7]. There are no specific regulations concerning the research participation of patients with psychiatric or addictive disorders. Proxy consent in research will be necessary if (a) persons are younger than 18 years of age (except for emancipated minors who are regarded as capable of making decisions); (b) the physician responsible ascertains that the patient's ability to take part in the decision-making process is impaired; or (c) the patient is legally incompetent [5, 6]. A person is considered legally incompetent if he is unable to understand or communicate information to meet essential requirements of physical health, safety or property management. In Spain, the courts are responsible for determining the legal competence of an individual basing their judgement on two medical reports.

As defined in psychiatric classifications, addiction is a disorder in which an individual’s control over their drug use is impaired [8]. People with an addiction continue to use drugs in the face of enormous negative consequences, and despite often expressing a wish that they could stop. This perspective is codified in the diagnostic criteria for addiction, in which a loss of control over drug use is central and becomes compulsive-something engaged in at the expense of all other goal-directed activities such as work or relationships [8]. Concerns have been raised about the capacity and voluntariness of people with SUD to participate in research. Some ethicts and clinicians have interpreted the DSM-5 criteria that describe loss of control and compulsive behavior in absolute terms [9, 10]. They argued that people with SUD fail to satisfy the required standards for competent voluntary consent and that we should assume that addicts are incompetent to consent to trials unless proven otherwise. Besides these caveats, there are other factors that can affect the ability of addicted individuals to provide consent to research because of the direct effects of their substance abuse as well as a wide range of co-morbid conditions. Acute drug intoxication or withdrawal may impair attention or retention of important information. Limited educational opportunities, chronic brain changes resulting from long-term drug use, poor nutrition, and co-morbid health problems are common in individuals with SUD and may also reduce concentration and limit understanding during the informed consent process [11].

Given the enormous health, economic and social burdens arising from SUD, there is strong public interest in preventing drug use [12]. The Demand Reduction Section of the United Nations International Drug Control Programme covers actions focusing in treatment and prevention, demand and supply reduction, international cooperation, training and improvement of scientific knowledge about SUD [13]. The Spanish National Drug Strategy 2009–2016 [14] is closely linked to the actions arising from the European Union and the United Nations (UN). The Spanish government has long cooperated in the policy and decision making bodies within the UN system, by providing technical assistance and funding for projects executed by bodies specializing in drugs [14]. Research in this field will lead to develop more effective treatments that will reduce the harm caused to the individual and society. Addicted individuals arguably have the same right to participate in, and benefit from, scientist research into their condition as anyone afflicted by any other disorder [15]. The potential personal, social and scientific benefits of neuroscience research of addiction, however, are not sufficient to justify research if it exploits a vulnerable population [16]. We need to show that those participating in such research are capable of consenting freely, that consent is obtained in ways that respects their autonomy, and that there is an acceptable balance of risk and benefit to addicted research participants.

For all these reasons the capacity assesment in addicted people becomes so important. The available data on decisional capacity among persons suffering from SUD are meager; very little research has focused on their comprehension of consent forms [17–19]. There is a need for studies of decisional capacity for consent to research among people with SUD, using standardized instruments [19]. Although there is no “gold standard” method for assessing capacity, the MacArthur Competence Assessment Tool for Clinical Research (MacCAT-CR) appears to be the single most widely used instrument for formal assessment of capacity to consent to research [20].

In 2013, a Spanish translation of the MacCAT-CR was made available by Baon [21] and in 2014; a manual was published to provide the assessing clinicians or researchers a structured method to aid them in the IC procedure [22]. However its use isn’t extended yet and evaluations of a patient’s decision-making competence are still based on intituive assesments.

The aim of the present study is to asses the capacity of addicted individuals to provide consent to research by means of the recent Spanish version of the MacCAT-CR.

## Methods

### Type of study

We conducted a cross-sectional survey, which was approved by the ethical research committee of Sta María del Rosell Hospital in Cartagena, Health Service of Murcia.

### Participants

Participants were 53 individuals seeking treatment for alcohol and/or illicit substance use at a community-based Outpatient Treatment Center and 50 non psychiatric comparison subjects (NPCs) from an urban-located health centre. The study was carried out over a 4-month period (From December 2014 to March 2015) in south-eastern Spain. Every consecutively referred or identified patient meeting the inclusion criteria was invited to participate. Participants included outpatients with DSM-5 diagnoses of SUD and NPCs diagnosed of hypertension, diabetes mellitus or other mild medical illnesses. Inclusion criteria were (a) currently 18 years or older, (b) diagnosis matching targeted conditions, (c) fluency in Spanish, (d) current score on the Spanish version of the Mini-Mental State Examination: (MMSE) 20 or higher [23], and (e) voluntary informed consent to participate in this study.

NPCs were excluded if (a) they met criteria for a DSM-5 current SUD or other Axis I diagnoses, (b) they were active patients at Mental Health Centre or at Center for Substance Abuse Treatment and (c) they were in psychiatric treatment with their General Practitioner.

Substance abusers were excluded if they were intoxicated or suffering acute withdrawal symptoms at the time when consent is requested.

### Measures

Patient information was collected through the use of a questionnaire designed to obtain data on variables regarding patient demographics and clinical characteristics. Level of functioning was evaluated using the Global Assessment Functional Scale (GAF) (addict subjects only), [24] and severity of symptoms was assessed using the Clinical Global Impression Scale (CGI) (addict subjects only) [25].

Judgments on mental capacity were based on a clinical assessment (review of notes and clinical interview) and the administration of the Spanish version of the MacCAT-CR [21]. This instrument is a semistructured interview adapted to the elements of a specific research protocol and it evaluates the 4 commonly recognized dimensions of decisional capacity: (a) *understanding* the relevant information; (b) *appreciation* of the effects of research participation on the patient’s own situation; (c) *reasoning* with the information in a decisional process and (d) *expressing a choice* about participation [20]. MacCAT-CR administration involves disclosure of information about the study that subjects are being asked to consider, in this case a hypothetical medication trial designed by ourselves, followed by questions that assess the four dimensions of decisional capacity. Each ability is assessed by specific questions with answers rated on a 0–2 scale with higher scores reflecting better performance. The understanding scale has thirteen questions (range 0–26), the appreciation scale has three questions (range 0–6), the reasoning scale has four questions (range 0–8) and the expressing a choice scale has only one question (range 0–2). This instrument has been widely used in research and is described in detail elsewhere [20, 26].

The MacCAT-CR does not yield a limit score on the four abilities, but for practical purposes in some studies, cut-points have been determined to identify those who lack capacity [26–29]. Previous studies in populations with dementia or psychiatric disorders have demonstrated a high degree of reliability [30] and indications of validity [21, 31]. An Understanding score of 20 or higher on the 26-point scale was required as a minimum for being capable, according to the study of Carpenter although clinical judgment was the final determinant of competence to consent even if a subject achieved this threshold [26]. This threshold reflected an a priori judgment by the investigators of what constituted minimally adequate understanding of this specific research protocol. The understanding scale was used to establish the threshold of capacity because understanding generally correlates highly with appreciation and moderately with reasoning, and has the strongest psychometric properties of the three scales [32].

### Procedures

We used an interactive consent process to ensure adequate understanding of the protocol basics. A research assistant met with the potential participants (together with their legal guardians if they were legally incompetent) and gave them both verbal and written information about the study. Cognitive state was evaluated by using the MMSE excluding those patients with advanced cognitive impairment. The research assistant reviewed the information in the consent form with the potential participants, and then, written informed consent was obtained from all patients and from legal guardian if the participant was legally incompetent. No subject was legally incompetent, so this surrogate consent procedure wasn’t necessary.

Within one month following the initial assessment, two trained research assistants conducted all the capacity evaluations. Research technicians were trained by the research coordinator to administer the MacCAT-CR in a scripted and highly standardized manner. The research coordinator conducted multiple role plays of the consent process with the research technicians until they had successfully mastered the process.

We developed a hypothetical consent form describing a randomized controlled trial of an experimental compound (which we named *“Semoca”*) being tested for hedeache, which was modeled after phase II studies of similar agents. The consent form described a 16-week randomized comparison of Semoca vs placebo. It was 5 doubled-spaced pages and had a Flesch-Kincaid reading level of 13, the reading level generally suggested in the literature [33]. Procedures described to the patients included randomized assignment, blinded exposure to a new tablet, the voluntary nature of participation, procedures for withdrawal from the study and risks (blood draw and non-life-threatening side effects of the drug). The inability to guarantee direct benefit was explained as well. Each subject read this hypothethical project aloud and then, the MacCAT–CR was administered. We stopped the interview if there was any change in choice, or resistance. Only one patient was lost.

After each interview, the researcher scored the four subscales according to MacCAT–CR criteria. The interviews weren’t videotaped but there was a verbatim transcription because the investigators interviewed the subjects with other co investigator present who took notes. Later on, these transcribed interviews were independently reviewed by two people (a medical ethics specialist and a psychiatrist) each of whom assigned their own scores to each patient based on the MacCAT-CR rating guidelines. A consensus meeting was then held to derive a single score for each case when the judgment was felt to be difficult. In practice this amounted to seven interviews. The research team made a global judgement about the patient’s capacity to consent to research, based on information from both the MacCAT–CR and the clinical interview with the patient. The investigators met regularly with I.M.S. and M.D.P.C. to review and discuss any scoring questions.

### Data analysis

Data were analyzed with SPSS (version 19.0). Prior to analysis, all data was examined for normality and homogeneity of variance. Differences between groups on ordinal or continuous data were analyzed using the two-sample parametric *t* test/1-way analyses of variance and the Mann–Whitney *U* test/ Kruskal- Wallis test for non parametric data. Group differences in categorical variables were compared with using Pearson’s χ2 or Fisher’s exact test for non parametric data.

To examine the relationship between the independent and dependent variables, we calculated the odds ratios (ORs) and 95 % CIs. Multivariate analyses with logistic regression were carried out with the variables that showed a significant relation with the dependent variable in the univariate analyses and were clinically relevant. To avoid an overfit model we followed the rule which states that for every independent variable, there should be no fewer than 10 events per covariate [34]. Then, by direct selection, we obtained a model with the individual variables directly related to the dependent variable (lack of capacity). To assess model’s overall fit to the sample data we used the Hosmer-Lemeshow goodness-of-it test. It produced a high p-value so our model passed the test and the difference between observed and model-predicted values was small. Colinearity tests were carried out between explanatory variables, calculating the variance inflation factor (VIF) for each. This gave a value of VIF < 3 for each variable, so colinearity was rejected, meaning that the possibility of overfitting was minimal. A probability level of p ≤ 0.05 (two tailed) was considered significant.

## Results

### Demographic and clinical characteristics

**Fig. 1:**
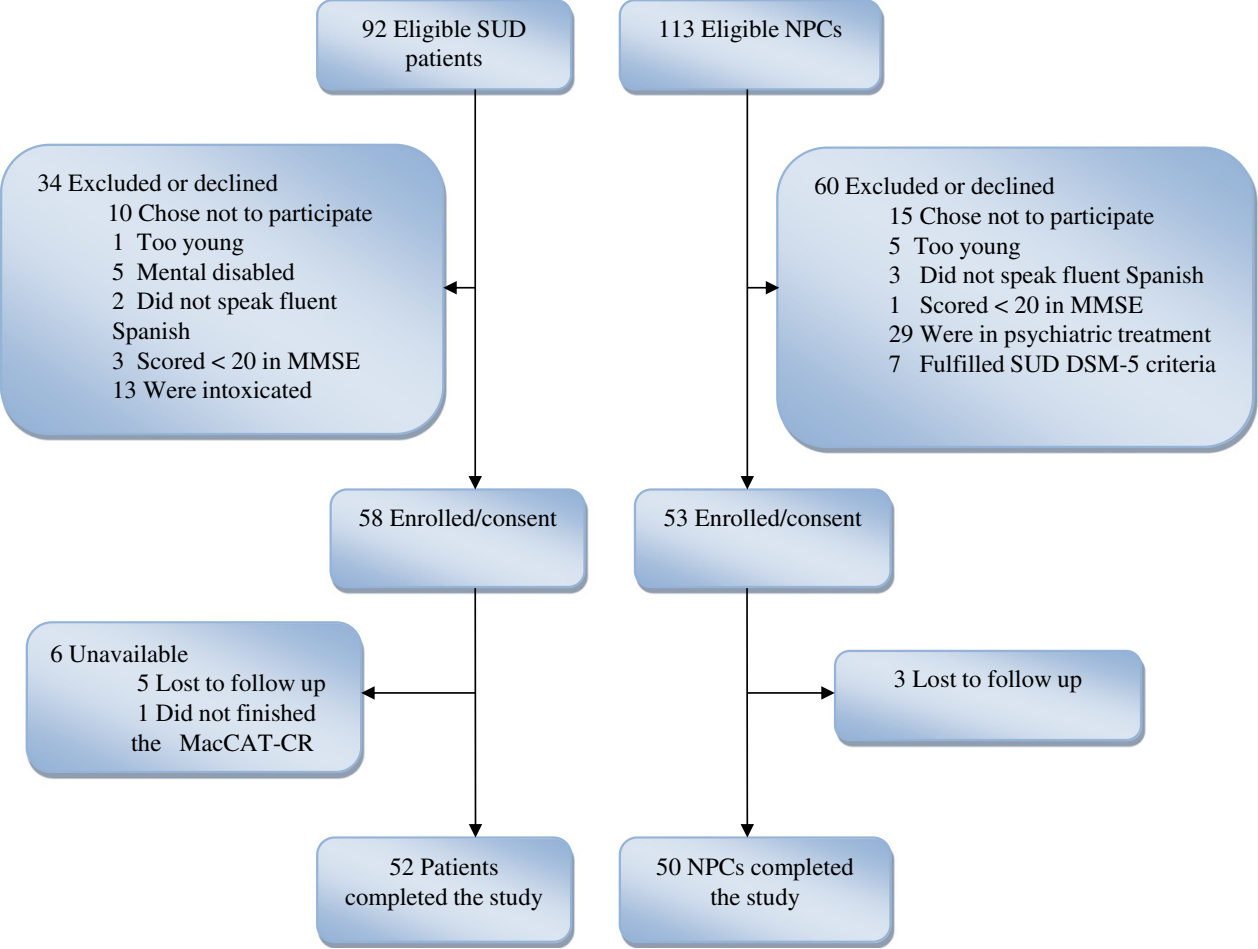
Flowchart of participant’s inclusion. Abbreviations: SUD, Substance use disorders; NPCs, Non psychiatric comparisons

Of the 205 subjects eligible for this study, 103 were excluded or were unavailable to participate for various reasons (Fig. 1).

The SUD group was the youngest 42.9 ± 11.9 years (*p* < .001). The proportion of women was highest among NPCs 62.0 % (*n* = 31) vs 28.3 % (*n* = 15) (*p* < .001). Most NPCs 88 % (*n* = 41) were married whilst 67.9 % (*n* = 36) SUD people have never married or have been previously married (*p* < .001). 88 % NPCs (*n* = 41) live with their family and none live in an institution while 45.3 % SUD people (*n* = 24) live with their families and 39.6 % (*n* = 21) with their parents or in an institution (*p* < .001). The patients with SUD had fewer years of education than those in the other group (*p* < .001), only 9.4 % SUD people (*n* = 5) completed university degree vs 40 % NPCs (*n* = 20). Most of NPC were working 84 % (*n* = 42) vs 20.8 % (*n* = 11) SUD people (*p* < .001). The SUD group had the most severe cognitive deficits, lowest mean MMSE total score, 28.2 ± 4.2 points (*p* < .001).

Baseline characteristics of each SUD group are described in Table 1. The 53 patients with SUD had the following diagnoses: 45.3 % (*n* = 24) had and alcohol or cannabis use disorder, 18.9 % (*n* = 10) had a cocaine use disorder and 35.8 % (*n* = 19) used alcohol and other drug.

**Table 1:** Baseline characteristics of each SUD group

Variable	Alcohol	THC	Cocaine	Alcohol + other	*P* Value
	(*n* = 12)	(*n* = 12)	(*n* = 10)	(*n* = 19)	
Age, years ( mean ± SD)	51.7 (7.6)	32.9 (12.7)	43 (10.8)	43.6 (9.7)	.003^b^
Women. (%)	33.3	25.0	20.0	31.6	.201^a^
Marital Status (%)					
Married/cohabiting	33.3	25.0	30.0	36.8	.708^a^
Never married	41.7	58.3	50.0	36.9	
Previous married	25.0	16.7	20.0	26.3	
Living status (%)					.469^a^
Alone	25.0	8.3	0.0	21.1	
With family	50.0	41.7	40.0	47.3	
Parents/institution	25.0	50.0	60.0	31.6	
Education level (%)					.975^a^
Primary	58.3	50.0	70.0	57.9	
Secondary	33.3	41.7	20.0	31.6	
University degree	8.3	8.3	10.0	10.5	
Employment status (%)					.540^a^
Employed	25.0	8.3	0.0	36.8	
Unemployed	41.7	50.0	50.0	42.1	
Retired	16.7	16.7	20.0	5.26	
Disabled person	16.7	25.0	30.0	10.5	
Psychiatric diagnosis (%)					.080 ^a^
Psychotic disorders	0	33.3	20	15.8	
Mood Disorders	50	16.7	10	26.3	
Anxiety disorders	25	50	20	31.6	
No psychiatric diagnosis	25	0	50	26.3	
CGI (%)					.926^a^
Less than moderately ill	16.7	25.0	20.0	26.3	
Moderately ill or more	83.3	75.0	80.0	73.7	
GAF (range, 0–100) ( mean ± SD)	65.8 (12.8)	66.3 (19.2)	64.4. (14.8)	67.6 (15.9)	.879^b^
Length of illness, years( mean ± SD)	19.7 (13.3)	7.4 (7.1)	9.8 (6.6)	18.1 (9.1)	.004^b^
Psychiatric Admissions (mean ± SD)	0.6 (0.8)	0.7 (1.4)	0.6 (0.8)	1.7 (3.4)	.995^b^
Therapeutic Community Admisisons (mean ± SD)	0.25 (0.4)	0.08 (0.3)	1.1 (1.5)	0.37 (0.6)	.106 ^b^
MMSE (range, 0–30) ( mean ± SD)	28.5 (1.6)	27.0 (8.5)	29.0 (1.5)	28.4 (1.6)	.765^b^

*Abbreviations*: SUD, Substance use disorder; THC, Tetra hidro cannabinol; SD, standard deviation; CGI, Clinical Global Impression; GAF, Global Assessment of Functioning; MMSE, Mini-Mental State Examination.

^a^ Pearson’s χ2

^b^ Mann–Whitney U

### Decisional capacity characteristics

Decisional capacity characteristics of each group are described in Table 2.

**Table 2:** Decisional capacity characteristics

Variable	Substance Use Disorders (*n* = 52)	Non psychiatric comparison subjects (*n* = 50)	*P* Value
Capacity (%)	67.3	84.0	.05 ^b^
MacCAT-CR ( mean ± SD)			
Understanding subscale score (range, 0–26)	20.1 (5.2)	23.0 (3.5)	<.001^a^
Appreciation subscale score (range, 0–6)	5.0 (1.4)	5.7 (0.8)	.003 ^a^
Reasoning subscale score (range, 0–8)	6.1 (1.8)	6.5 (1.4)	.359 ^a^
Expression of a choice subscale score (range, 0–2)	1.8 (0.5)	2.0 (0.2)	.159 ^a^

*Abbreviations*: SD, standard deviation; MacCAT-CR, MacArthur Competence Assessment Tool for Clinical Research

^a^ Mann–Whitney U

^b^ Pearson’s χ2

On the MacCAT-CR subscales patients with SUD garnered the lowest scores, particularly on the Understanding and Appreciation subscales: 20.1 ± 5.2 (*p* < .001) and 5 ± 1.4 (*p* = 0.03) points respectively.

**Fig. 2:**
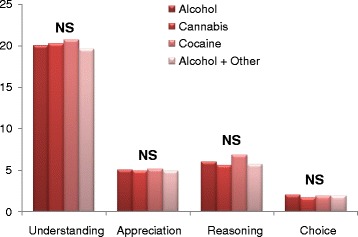
MacCAT-CR scales scores in SUD. Abbreviations: MacCAT-CR, MacArthur Competence Assessment Tool for Clinical Research; SUD, Substance Use Disorders; NS, Non significant

Differences on MacCAT-CR subscales scores within each SUD group are illustrated in Fig. 2. There were no statistically significant differences between the groups.

### Ratings and factors linked with incapacity

32.69 % of the patients (*n* = 17), lacked research-related decisional capacity, based on a judgement guided by the MacCAT–CR and a clinical interview.

Socio-demographic and clinical and decisional capacity characteristics of patients with and without mental capacity are shown in Table 3.

**Table 3:** Comparison of patients with and without capacity to consent to research

Variable	Capacity present	Capacity absent	*P* Value
	(*n* = 35)	(*n* = 17)	
Age, years (mean ± SD)	42.3 (11.3)	44.9 (11.2)	.545^a^
Women (%)	86.7	13.3	.045 ^b^
Men (%)	59.5	40.5	
Marital Status (%)			.239 ^b^
Married/cohabiting	75	25	
Never married	78.6	21.4	
Previous married	54.5	45.5	
Living status (%)			.041 ^b^
Alone	75	25	
With family	75	25	
Parents/Institution	55	45	
Education level (%)			.033 ^b^
Primary	55.3	46.7	
Secondary	82.4	17.6	
University degree	100	0	
Employment status (%)			.198 ^b^
Employed	72.7	27.3	
Unemployed	52.2	47.8	
Retired	85.7	14.3	
Disabled person	81.8	18.2	
Psychiatric diagnoses (%)			.668 ^b^
Psychotic disorders	55.6	44.4	
Mood Disorders	78.6	21.4	
Anxiety disorders	62.5	37.5	
No psychiatric diagnosis	69.2	30.8	
CGI (%)			<.006 ^b^
Less than moderately ill	100	0	
Moderately ill or more	57.5	42.5	
GAF (range, 0–100) (mean ± SD)	70.2 (16.7)	57.6 (8.1)	.003 ^a^
Length of illness, years (mean ± SD)	12.9 (10.1)	18.5 (10.5)	.056 ^a^
Psychiatric Admissions (mean ± SD)	0.9 (1.5)	1.4 (3.4)	.782 ^a^
Therapeutic Community Admisisons (mean ± SD)	0.26 (0.6)	0.75 (1.1)	.058 ^a^
MMSE scores (range, 0–30) (mean ± SD)	29.1 (1.3)	28.1 (1.4)	.007 ^a^
Group (%)			.435 ^b^
Alcohol	50	50	
THC	81.8	18.2	
Cocaine	70	30	
Alcohol + other	68.4	31.6	
MacCAT-CR ( mean ± SD)			
Understanding subscale score (range, 0–26)	23.0 (1.9)	15.2 (3.3)	<.001 ^a^
Appreciation subscale score (range, 0–6)	5.4 (1.0)	4.3 (1.5)	.008 ^a^
Reasoning subscale score (range, 0–8)	6.7 (1.2)	5.1 (1.9)	.002 ^a^
Expression of a choice subscale score (range, 0–2)	1.9 (0.2)	1.8 (0.6)	.164 ^a^

*Abbreviations*: SD, standard deviation; CGI, Clinical Global Impression; GAF, Global Assessment of Functioning; MMSE, Mini-Mental State Examination; THC, Tetra hidro cannabinol; SUD, Substance use disorders; MacCAT-CR, MacArthur Competence Assessment Tool for Clinical Research

^a^ Mann–Whitney U

^b^ Pearson’s χ2

The relationships between patients characteristics and lack of capacity are the following: using male gender as a reference, the OR of lack of capacity was 4.43 (IC95%: 1.27-8.52*; p* = 0.044) for the women of our study. Thus, in our sample, men seemed to be more likely to lack capacity than female patients. Each one-point increase in the MMSE score increased the chance of being capable by approximately 6 %: OR = 0.60 (IC95%: 0.39-0.93; *p* = 0.02).

The logistic regression model included relevant variables that were significantly associated with lack of capacity on univariate analysis. We choose MMSE scores by its statistical performance and because impaired cognition is well-recognized as a limitation to the research participation. Gender was selected by its apparent high OR. Only one of the two variables included in the univariate analysis was retained in the multivariate model: the MMSE scores: OR = 0.602 (IC95%: 0.38– 0.95; *p* = 0.029).

When other variables associated with lack of capacity on our univariate analysis are considered, gender no longer has an impact on the probability of being capable OR = 4.36 (IC95%: 0.81-8.06; *p* = 0.86).

## Discussion

We believe this study to be important because is the first one that specifically assess the capacity to provide consent to research among people with SUD by means of a standardized instrument such as the MacCAT-CR. Most people diagnosed of SUD in our survey were able to provide consent to research, we didn’t find statistically significant differences between NPCs and SUD groups in terms of capacity to consent to research.

Patients with SUD showed the worst performance on the measures of the MacCAT-CR, especially in the Understanding and Appreciation dimensions. Given the contextual nature of decisional capacity, as well as the characteristics of the MacCAT-CR interview, a comparison of results across studies is somewhat difficult. The scale does not provide cut-off scores nor is there an algorithm for categorical determinations of capacity or incapacity. General consensus exists that as the degree of risk increases, a higher level of capacity is desirable. This is appropriate, as studies vary in level of risk and in the risk/benefit ratio. Therefore, there is not a particular level of ability which represents adequate capacity in all circumstances [35]. Mean levels of MacCAT-CR scores vary widely within different disorders. The mean Understanding total score in our SUD group was 20.1 points; and 5.0 points on the Reasoning dimension. We didn’t find previous research about SUD and MacCAT-CR performance to compare with. Our findings will have to be explored in future studies.

There is much variability in addicted individuals’response to drugs and the degree of impairment they experience. As literature suggests, impairment may differ depending on the type of drug being abused, the route of administration, the severity of the addiction, the level of tolerance, and the amount of time since last drug use [12, 36]. Unexpectedly, we didn’t find significant differences between SUD groups in our study in terms of lack of capacity. The limited number of patients included in each SUD group could be one explanation for these results.

A non- abstinent drug-dependent individual can spend significant time between periods of acute intoxication and withdrawal in which they are not severely cognitive impaired. Addiction may therefore only affect addicted individuals’ability to consent to research in some individuals and in some situations. This impairment is not absolute, and is not seen in all individuals in all circumstances at all times. It is unwise to assume that decision-making in addicted persons is so impaired to eliminate autonomy [18].

In the present study we found an association between education level and mental capacity. This may be due to some factors such us cognitive decline [37]. The chronic use of addictive drugs can cause significant cognitive deficits that may impair an addicted person’s capacity to provide informed consent. Yet while comparison of absolute levels across studies is difficult, the importance of cognitive functions in a decisional capacity is a very consistent finding across multiple disorders [37].

Our findings indicate that men were more likely to lack capacity than female patients. Although gender generally has little demonstrable influence on cognitive tasks [38], we don’t know specific studies about the influence of gender in research- decision-making capacity. One study examined the level of performance of women with major depression on the decision-making abilities assessed by the MacCAT-CR but they didn’t include male participants to compare the scores with [39]. In our study when the other variables associated with incapacity on the multivariate analysis are considered, gender has no longer a significant impact on the probability of being capable.

Mental capacity is also univariately associated in our study with living status. People who live with their parents or in an institution were more likely to lack capacity than those who live alone or with their families, although we didn’t confirm this association in the logistic regression model. As literature suggests, living status may be linked to lack of capacity: given that the level of functionality corresponds to competence, is it possible that living alone reflects functionality [40]. The chronic use of drugs can cause significant cognitive deficits that may impair addicted people’s capacity to take care of themselves or to live by their own so is more difficult for people with SUD to achieve emancipation.

Most patients in our study have also a psychiatric disorder. In Spanish substance misuse services up to 70 % of patients have co-morbid psychiatric disorders [41].These patients with dual diagnosis may have dual deficits in decisional capacity— compounded by impaired response to short-term versus long-term gains and losses and those secondary to cognitive impairment associated with psychiatric disorders that can influence the decisional process [19]. However, we didn’t find significant differences between patients with and without dual diagnoses in our study in terms of lack of capacity. These results will have to be explored in further research.

Symptom severity, as measured by GAF and CGI scores was associated with lack of capacity in this research. Our findings suggest that the dual diagnosis severity, as measured by lower social functioning may be a factor in the decisional process.

Only one of the considered variables remained independently associated with incapacity on multivariate analysis: the cognitive state. These results indicate that cognition must be considered in capacity assessment, regardless of group as literature suggests [37].

Our results highlight the importance of assessing the capacity of research participants to provide consent. It is widely held that subjects are deemed competent, unless it can be proved otherwise, to participate in the informed consent process. If the participants do not fully comprehend the procedures they are consenting, safeguards should be implemented to protect them, but we argue these safeguards should be tailored capacity-based not diagnoses-based. The view that addicted individuals lack the capacity to give a free informed consent by the virtue of being addicted, would, if accepted, significantly hinder research on addiction, and hence the development and evaluation of new treatments that may benefit addicted persons. This would not only be a violation of the principle of justice (equal distribution of the benefits of research) but it may lead to poorer outcomes for addicted individuals and society [18]. This approach to people with SUD raises ethical concerns about respect for the principle of autonomy. Increased restrictions on research involving persons with SUD, no matter how well intentioned, may reflect and reinforce stigmatization of those persons. Beyond the diagnosis, other sets of factors have been identified as influencing the process capacity determination: the risk context, and the knowledge and characteristics of those making the judgment about decision-making capacity [36, 42]. An evaluator who places greater value on protecting an individual from potential harm may, for any given risk–benefit scenario, require a higher threshold of ability than another evaluator who tends to place on the side of allowing the person to determine his or her own course. This apparently enhanced sensitivity to human subject’s protection in SUD research may overestimate the vulnerability of SUD research participants.

Assesment of competence must be regarded as risk-related and task-related: a specific judgement at a specific moment of the ability of one person to fulfill a concrete task. The application of this model has implications for designing a research protocol, especially when standards for competence are being set [36]. For example, it’s necessary that investigators state a priori judgment of what constituted minimally adequate understanding of a specific research protocol. The threshold for finding an individual capable of making a decision should vary depending upon the risks and benefits involved (i.e., when the stakes are higher, a higher level of ability is necessary).

As our results has shown, lack of understanding in informed consent process may occur both in clinical population and in NPCs, so we argue that routine evaluation of decisional capacity in greater-than-minimal-risk studies may be reasonable. Such screening is therefore just as appropriate when patients are physically ill as when they are mentally ill: there is no evidence which supports inferring a uniform ability or inability to make decisions on the basis of a specific diagnosis [43].

### Limitations

There are a number of limitations that should be considered when interpreting the results. First, our study was carried out in an urban setting and it was restricted to a limited number of outpatients which may limit the generalisation of the results. Further research is necessary to assess our results in other settings and participant groups. Studies with larger sample sizes would also allow investigators to conduct multiple-regression analyses with a greater number of independent variables in order to confirm all the univariate associations in the logistic regression model.

A second limitation arises from the cross-sectional data origin. The results presented here do not allow us to examine changes in consent-related abilities over time, nor to identify predictors of change. Consequently, prospective studies about SUD and capacity to consent to research should be conducted to identify those people that may require safeguards tailored to protect their rights.

Another limitation that should be considered is that both the non-random nature of the sample and the absence of other SUD (such as heroine and anxiolytics/hypnotics use disorders) raise questions about the generalizability of the results. Future studies should asses capacity in these SUD.

## Conclusions

The findings of our study provide evidence that a large proportion of individuals with SUD had decisional capacity for consent to research. It is therefore inapropiate to draw conclusions about capacity to make research decisions on the basis of a SUD diagnosis, because most of addicted people remain capable of giving autonomous consent under most circumstances. In the absence of acute withdrawal or intoxication or advanced cognitive impairment, we should assume that addicted persons possess decision-making capacity. Thus, the view that people with SUD would ipso facto lose decision-making power for research consent is flawed and stigmatizing.

Institutional Review Boards and investigators should consider these caveats as they decide which populations or individual subjects may require more intensive evaluation or further educational efforts to enhance decisional capacity, especially for greater-than-minimal-risk studies like our hypothetical study was.

## Abbreviations

CGI: Clinical Global Impression Scale
CI: Confidence intervale
DSM-5: Diagnostic and Statistical Manual of Mental Disorders
GAF: Global Assessment Functional Scale
MacCAT–CR: MacArthur Competence Assessment Tool for Clinical Research
MMSE: Mini-Mental State Examination
NPCs: Non psychiatric comparison subjects
OR: Odds ratio
SD: Standard deviation
SUD: Substance use disorders
THC: Tetra hidro cannabinol
UN: United Nations
VIF: Variance inflation factor
